# Emerging roles of circular RNAs in regulating the hallmarks of thyroid cancer

**DOI:** 10.1038/s41417-024-00736-0

**Published:** 2024-02-05

**Authors:** Tianjiao Zhou, Zheng Li, Yumeng Jiang, Kaiming Su, Chuan Xu, Hongliang Yi

**Affiliations:** 1 Department of Otorhinolaryngology Head and Neck Surgery, Shanghai Sixth People’s Hospital Affiliated to Shanghai Jiao Tong University School of Medicine, Shanghai, 200233 China; 2 Shanghai Key Laboratory of Sleep Disordered Breathing, Shanghai, 200233 China; 3 Otolaryngology Institute of Shanghai Jiao Tong University, Shanghai, 200233 China; 4 Bio-X Institutes, Key Laboratory for the Genetics of Developmental and Neuropsychiatric Disorders, Ministry of Education, Shanghai Jiao Tong University, Shanghai, 200240 China

**Keywords:** Gene regulation, Thyroid cancer, Thyroid cancer

## Abstract

Thyroid cancer is a prevalent endocrine malignancy with increasing incidence in recent years. Although most thyroid cancers grow slowly, they can become refractory, leading to a high mortality rate once they exhibit recurrence, metastasis, resistance to radioiodine therapy, or a lack of differentiation. However, the mechanisms underlying these malignant characteristics remain unclear. Circular RNAs, a type of closed-loop non-coding RNAs, play multiple roles in cancer. Several studies have demonstrated that circular RNAs significantly influence the development of thyroid cancers. In this review, we summarize the circular RNAs identified in thyroid cancers over the past decade according to the hallmarks of cancer. We found that eight of the 14 hallmarks of thyroid cancers are regulated by circular RNAs, whereas the other six have not been reported to be correlated with circular RNAs. This review is expected to help us better understand the roles of circular RNAs in thyroid cancers and accelerate research on the mechanisms and cure strategies for thyroid cancers.

## Introduction

Thyroid cancer (TC) is a prevalent endocrine malignancy affecting approximately 586,000 individuals, resulting in 44,000 fatalities globally in 2020 [1]. Radiation is an environmental factor that elevates the risk of developing TC [2]. TC affects mostly females, and most thyroid cancers are discovered through ultrasound without specific clinical symptoms. After surgical treatment also, thyroid cancers have a recurrence rate as high as 10% [3]. Metastatic, locally advanced, and iodine-refractory thyroid cancers present formidable clinical challenges [4]. For instance, the median overall survival of patients with anaplastic TC (ATC) is only 0.79 years [5]. Therefore, a thorough investigation of the fundamental mechanisms underlying the pathogenesis of thyroid cancers is imperative to elucidate the intricate factors contributing to the varied clinical prognostic outcomes.

“The Hallmarks of Cancer” thoroughly explores the progress and discoveries in cancer research, spanning nearly a century, presenting a coherent framework for comprehending the varied phenotypes of cancers [6]. The third edition in 2022 expanded the number of cancer hallmarks to 14, from the previous eight presented in the second edition in 2011 [7], by introducing four new hallmarks: “unlocking phenotypic plasticity,” “non-mutant epigenetic reprogramming,” “polymorphic microbiome,c” and “senescent cells.” [8] Most of these hallmarks have been validated, inspiring investigations into their underlying mechanisms.

Non-coding RNAs (ncRNAs), once regarded as “non-functional,” have now been established as both oncogenic drivers and tumor suppressors across various major cancer types, providing a novel direction for understanding the diverse phenotypic manifestations of cancer [9–12]. Circular RNAs (circRNAs), a distinct subtype of ncRNAs, are generated by alternative splicing and occurs widely found in cancers. For instance, the CSCD-2.0 database has unveiled over a million circRNAs in human cancers [13].

CircRNAs play significant regulatory roles in cancers [13–15], promoting or inhibiting cancer development. Notable examples include circRHOBTB3, circCDYL, and circLRFN, which have been implicated in promoting colorectal cancer [14–16], breast cancer [17–19], and glioma [17, 18, 20, 21] progression through a range of molecular mechanisms, including functioning as miRNA sponges, interacting with RNA-binding proteins (RBPs), and encoding proteins. CircRNAs exhibit similar roles in TC: for example, hsa_circ_100721 and has_circ_0001018, have been shown to promote the proliferation or metastasis of thyroid cancers by regulating epithelial-mesenchymal transition (EMT) or the cell cycle [19, 22]. CircRNAs also mediate the molecular regulation of TC hallmarksat the molecular level, including sustaining proliferative signaling, evading growth suppressors, activating invasion and metastasis, inducing angiogenesis, resisting cell death, deregulating cellular energetics, regulating non-mutant epigenetic modifications, and unlocking phenotypic plasticity. However, the role of circRNAs in the other six hallmarks of TC remains largely unexplored.

Our aim was to summarize the emerging roles of circRNAs in thyroid cancers based on the latest 14 cancer hallmarks, by reviewing the existing studies, with the intention of encouraging further experimental research into the biology, genetics, and pathogenesis of circRNAs.

## Overview of functional circRNAs in TCs

**Fig. 1:**
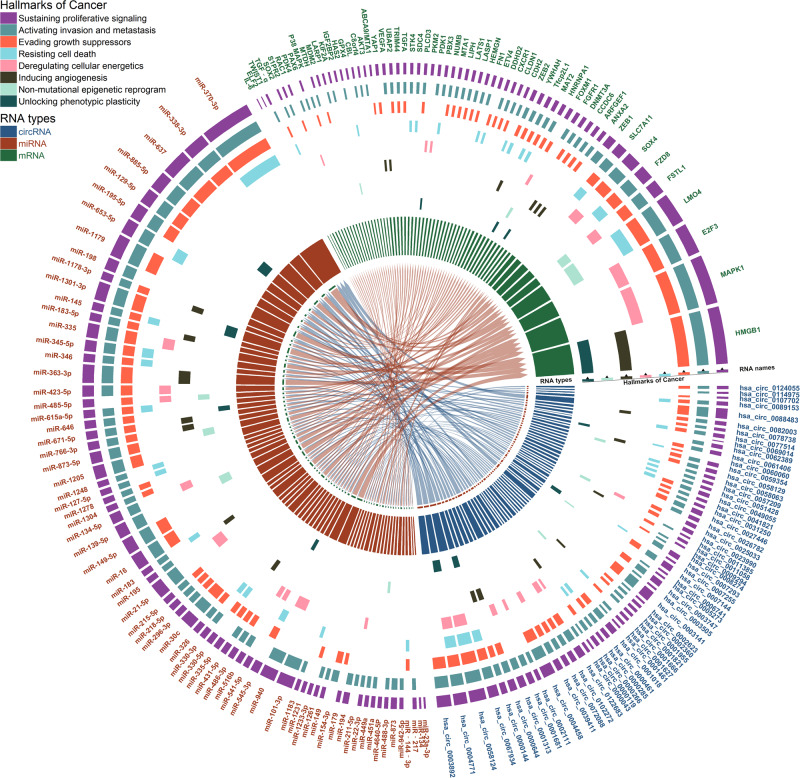
CeRNA mechanism of circular RNAs in thyroid cancer research involving miRNA and mRNA interactions, with a focus on hallmarks of cancer. In this depiction, the concentric circles from outermost to innermost correspond to the following elements: (1) RNA names, (2–9) hallmarks of cancer, (10) RNA types, and (center) the interaction network.* The angular size of each sector represents the total number of interactions involving this RNA.

We retrieved English-language articles from PubMed and EMBASE databases until June 2023, focusing on TC and circular RNAs, following MESH list. We excluded reviews, bioinformatics analyses, and non-experimental researches. By PMID numbers, we synthesized the characteristics of circRNAs, their molecular mechanisms (target genes or proteins), involvement in cancer hallmarks, pathology, and AJCC TMN stage (Supplementary Table 1). Finally, we retrieved and analyzed 100 research articles, reporting 71 TC circRNAs, visualized by the Circlize package in R [23] (Table 1 and Fig. 1). CircRNAs has shown that the majority of pathological types are papillary TC (PTC), with a small number being poorly differentiated TC (PDTC) or anaplastic TC (ATC). CircRNAs that promote TC progression also exhibit higher expression levels in advanced AJCC TMN stage. Compared with adjacent non-cancerous tissues, we found that most circRNAs (66 out of 71) were upregulated, while only five were downregulated. There TC circRNAs could be categorized into three types: exon circRNAs (eciRNAs), exon-intron circRNAs (elciRNAs), and intron circRNAs (ciRNAs). EciRNAs derived solely from exons were the most common, accounting for 66 out of the 71, while only four and one were ciRNAs and elciRNAs, respectively. The other two specific subtypes, intergenic circRNAs located outside known gene loci and antisense circRNAs overlapping with the linear RNA gene locus but are transcribed from the opposite strand, have not yet been reported in thyroid cancers.

**Table 1:** Characteristics of circRNA researchs in thyroid cancer.

Characteristics	Item	Number
Research articles		**100**
Pathology	PTC	85
	PDTC/ATC	2
	Unclear	13
Relative exp by III-IV/I-II stage	Higher	38
	lower	4
	Unclear	58
CircRNA		71
Expression		
	Up-regulated	66
	Down-regulated	5
Type		
	EciRNA	66
	CiRNA	4
	ElciENA	1
	Antisense circRNA	0
	Intergenic circRNA	0
Mechanisms		
	Sponge miRNA	67
	RNA-binding proteins	1
	Unclear	3
Target gene		66
Signal pathway		
	PI3K/AKT	9
	MAPK	6
	Wnt/β-catenin	4
	mTOR	2
	AMPK	2
	Hippo	1
	Notch	1
Hallmarks		
	Sustaining proliferative signaling	70
	Evading growth suppressors	46
	Avoiding immune destruction	0
	Enabling replicative immortality	0
	Tumor-promoting inflammation	0
	Activating invasion and metastasis	63
	Inducing angiogenesis	7
	Genome instability andmutation	0
	Resisting cell death	18
	Deregulating cellular energetics	9
	Unlocking phenotypic plasticity	4
	Non-mutational epigenetic reprogramming	4
	Polymorphic microbiomes	0
	Senescent cells	0

CircRNAs are linked to important regulatory functions, including acting as miRNA [24] or protein sponges to regulate gene expression [25], acting as scaffolds to mediate the formation of complexes [26], and potentially translating into small functional peptides [27]. In our collection of functional TC circRNAs, the majority operated through the miRNA sponge mechanism to regulate target genes, whereas only one circRNA was identified to interact directly with proteins. We did not find any circRNAs that regulate thyroid cancers through peptide translation. Notably, 66 target genes have been identified, associated with the regulation of TC circRNAs, primarily implicated in the PI3K/AKT, MAPK, Wnt/β-catenin, mTOR, AMPK, Hippo, and Notch signaling pathway.

The hallmark of cancer is the acquisition of functional abilities in human cells during the transition from a normal cell state to a tumor state, that contribute to cancer development. These hallmark characteristics alone do not fully elucidate the complexity of cancer pathogenesis (such as recurrence, metastasis, differentiation, and treatment resistance), and requires a multifaceted approach from various hallmarks. Among the 14 cancer hallmarks [8], eight have been investigated in relation to circRNAs. Among these, “Sustaining proliferative signaling,” “Evading growth suppressors,” and “Activating invasion and metastasis” are the first three most regulated. Nevertheless, six other hallmarks have not yet been linked to circRNAs. We reviewed all functional TC circRNAs associated with the eight hallmarks and explored prospects for future research regarding the other six hallmarks.

## CircRNAs and their roles in regulating TC hallmarks

The molecular functions of TC circRNAs include gene regulation in cancer-related pathways via binding to miRNAs or proteins. Given the well-established knowledge of cancer biological pathways, this article will delve into the introduction of circRNAs and their roles in each cancer hallmark, mainly by focusing on key circRNAs and their associated pathways/genes to maintain clarity and conciseness. Any circRNAs not introduced in detail below are shown in Fig. 1 and Supplementary Table 1. Although circRNAs reportedly influence the eight hallmarks, the research depths differ; thus, we also highlight the directions requiring further exploration.

### Sustaining proliferative signaling

The primary and fundamental hallmark of cancer is sustained proliferative signaling, which stimulates the uncontrolled growth of cells, finally leading to the development of tumors. Most of the collected circRNAs were linked to proliferative signaling in thyroid cancers, demonstrating their important roles in this hallmark.

Cancer cell proliferation predominantly depends on two signaling pathways: PI3K/AKT/mTOR and MAPK/p38. In thyroid cancers, circRNAs have been shown to influence cell proliferation by modulating these two pathways. In the PI3K/AKT/mTOR signaling pathway, has_circ_0009294 (circSSU72) competitively binds to miR-451a to upregulate S1PR2, resulting in the AKT phosphorylation [28], leading to proliferation of TC cells. Conversely, the knockdown of hsa_circ_0082003 (circPSD3) downregulates the phosphorylation of PI3K and Akt, thereby inhibiting TC cell proliferation. This inhibitory effect can be counteracted by either introducing a miR-637 inhibitor or overexpressing HEMGN [29]. Furthermore, AMPK, an important tumor suppressor kinase that inhibits mTOR [30], is regulatied by has_circ_0008274, that promotes the activation of the mTOR signaling pathway by inhibiting AMPK, thereby promoting the sustained proliferation of TC cells [31]. In the MAPK/p38 signaling pathway, knockdown of hsa_circ_0072088 (circZFR) suppresses TC cell proliferation, and this suppression is reversed upon MAPK overexpression [24]. Upregulation of circRNA NRIP1 reverses the inhibitory effect of miR-195-5p on the MAPK/p38 pathway in a nude mouse model of TC [32]. Additionally, there exists various other circRNAs and pathways related to proliferative signaling, as showed in Fig. 1 and Supplementary Table 1.

### Evading growth suppressors

In addition to growth factors, cells can generate growth suppressors, which function as stop signals for themselves or nearby cells if uncontrolled expansion threatens homeostasis. However, cancer cells are able to ignore these “anti-growth signals” and can continue to proliferate. This is a typical hallmark of cancer.

Specific signaling pathways related to p53 or E2F can disrupt the normal inhibition of cell proliferation, ultimately leading to cellular transformation into cancer cells. As a result, circRNAs that influence the expression of p53 or E2F may help cancer cells evade growth suppression. In thyroid cancers, a substantial number of circRNAs have been identified to play pivotal roles in enabling cancer cells to evade growth inhibition [33, 34]. For example, hsa_circ_0107702 (circTP53), formed by the reverse splicing of exons 5 to 9 of the TP53 gene, reduces p53 protein levels by targeting the miR-1233-3p/MDM2 axis, thereby promoting cancer cell proliferation [35]. Similarly, hsa_circ_0000644 [36], hsa_circ_0107702 [37], and hsa_circ_0031250 [38] were found to upregulate E2F expression, which influences entry into the S phase and apoptosis [39]. These circRNAs function as microRNA sponges and inhibit apoptosis, thereby promoting cancer progression.

### Activating invasion and metastasis

Cancer cells invade neighboring tissues and undergo metastasis, a hallmark of cancer that often leads to death. Cancer progression and metastasis involve the abnormal reactivation of epithelial-mesenchymal transition (EMT), a process regulated by Wnt/β-catenin, NOTCH, NF-κB, and other signaling pathways [40]. circRNAs regulate the expression of genes related to these pathways, either activating or inhibiting them, thereby influencing invasion and metastasis.

For example, hsa_circ_0004789 (circRNA_102171) can directly bind to the CTNNBIP1 protein, blocking its interaction with the β-catenin/TCF3/TCF4/LEF1 complex. Consequently, the Wnt/β-catenin pathway is activated, promoting EMT in TC [41]. Similarly, hsa_circ_0088483 (circNEK6) promotes TC metastasis by upregulating the expression of the key gene *FZD8* in the Wnt/β-catenin pathway [42]. Because NUMB can significantly inhibit the NOTCH pathway [43], hsa_circ_0058124 upregulates NUMB expression through a miR-218-5p sponge, thereby inhibiting the NOTCH pathway and promoting the invasion ability of thyroid cancers [44]. Moreover, it has been demonstrated that HMGB1 can bind to its receptor RAGE to promote EMT and invasion of cancer cells [45]. In thyroid cancers, hsa_circ_0062389 (circ0062389) competitively binds to miR-1179, resulting in upregulation of HMGB1 expression, inhibition of E-cadherin protein expression, and enhancing TC metastasis [46].

In addition, specific transcription factors such as ZEB1 and TWIST can influence multiple EMT-related signaling pathways [47]. Hence, circRNAs regulating these transcription factors through molecular interactions that affect EMT in cancers. For example, hsa_circ_0002623 (circVANGL1) [48] and hsa_circ_0001461 (circFAT1) [49] regulate ZEB1 expression through ceRNA mechanisms. Additionally, circ_0001681 competitively binds to miR-942-5p, resulting in the upregulation of TWIST1 expression [50]. Both hsa_circ_0002623 and hsa_circ_0001461 promote EMT in TC. Not only within cells, but has_circ_007293 within exosomes can also promote EMT in TC, which may help in the development of novel therapeutic strategies [51]. In summary, a multitude of circRNAs have been shown to influence thyroid cancers by modulating various signaling pathways, thereby impacting EMT to activate invasion and metastasis.

### Inducing angiogenesis

Angiogenesis is a fundamental event in tumor growth and metastasis, where vascular endothelial growth factor (VEGF) plays a key role in this process. [43]. Therefore, several inhibitors targeting the VEGF pathway, such as axitinib and sorafenib, have been utilized in the treatment of advanced thyroid cancers. However, their efficacies remain unsatisfactory. Although the role of circRNAs in tumor angiogenesis has been reviewed in other cancers, their involvement in TC angiogenesis remains largely unexplored [52]. Understanding the mechanism of circRNAs in TC-specific angiogenesis is crucial for identifying potential treatment strategies.

Among the circRNAs we gathered (Fig. 1), some regulated VEGF-dependent tumor angiogenesis in thyroid cancers. For example, hsa_circ_0001821 (circPVT1) enhances TC progression by targeting miR-195, subsequently increasing its expression [53]. Furthermore, hsa _circ_0011058 [54] and hsa_circ_0082003 [55] (circ0002111) indirectly promote the translation of angiogenic proteins (VEGFA and FGF) by upregulating YAP1 and HMGB1 in thyroid cancers, respectively, thereby promoting angiogenesis in TCs.

Another potential VEGF-independent regulation of tumor angiogenesis by circRNAs has been observed. In the tube formation assay, hsa_circ_0000144 (circ0000144) [56] and hsa_circ_005935449 [57] upregulated the expression of YWHAH and ARFGEF1, respectively, via ceRNA mechanisms. This upregulation promotes angiogenesis in thyroid cancers. However, additional validation and in-depth investigation are warranted to elucidate the precise mechanisms underlying the promotion of angiogenesis by circRNAs through the modulation of target genes for identifying potential treatment strategies in TC.

### Resisting cell death

Programmed cell death (PCD) is defined as controlled death of a cell and is advantageous to the life cucle of an organism; [58, 58, 59] however, when dysregulated PCD can cause cancer [58]. Therefore, understanding the mechanisms by which cancer cells resist PCD could aid in the development of therapies that effectively eliminate cancer cells [59]. In our collection of circRNAs, some were reportedly functional in resisting cell death in thyroid cancers (Fig. 1).

Apoptosis, one of the earliest studied forms of PCD, mainly involves the Bcl-2 family of proteins (e.g., Bax and Bcl-2). These proteins regulate the mitochondrial outer membrane permeability and release apoptotic signaling proteins through multiple signaling pathways. In thyroid cancers, 10 circRNAs have been reported to inhibit cancer cell apoptosis via ceRNA mechanisms [60–68]. For instance, circ_0057209 upregulates STK4 by acting as a miR-183 sponge to activate the Hippo pathway. This leads to an increase in Bcl-2 levels and a decrease in Bax protein levels, thereby promoting apoptosis [69].

Autophagy is another common mechanism of cell death. Dysregulation of autophagy-related genes, impaired lysosomal degradation of cytoplasmic proteins, and damaged organelles can cause cancer development [70]. Currently, only circEIF6 has been identified in promoting autophagy through the miR-144-3p/TGF-α axis, ultimately enhancing cisplatin resistance in thyroid cancers [71].

Ferroptosis is a form of cell death triggered by iron-dependent phospholipid peroxidation, to which mesenchymal cancer cells are highly susceptible [72]. The classical inhibitory pathway for iron-dependent cell death is the GSH-GPX4 signaling axis, in which GPX4 inhibits iron-induced cell death [73]. CircKIF4A upregulates GPX4 expression by suppressing miR-1231, inhibiting iron-dependent cell death in thyroid cancers [74]. Similarly, circ0067934 upregulates SLC7A11 expression by competing with miR-543-3p, thereby promoting GPX4 expression and inhibiting ferroptosis, leading to TC progression [75].

Other novel modes of cell death have recently been reported, including pyroptosis, necroptosis, and cytoproptosis. Although certain circRNAs (e.g., hsa_circ_0001836 [76], circNEIL3 [77], and has_circ_0007312 [78]) have been found to promote pyroptosis in some cancers, the role of circRNA-mediated pyroptosis in thyroid cancers remains largely unknown. While necropoptosis has been reported in TCs [79], studies on the circRNA-mediated progression of TC through necroptosis are still lacking. Additionally, the relationship between cuproptosis and ncRNAs in thyroid cancers has been analyzed [80, 81]; however, the involvement of circRNAs in thyroid cancers through cuproptosis remains unexplored.

### Deregulating cellular energetics

Cancer development involves not only the deregulated control of cell proliferation but also corresponding adjustments in energy metabolism to fuel cell growth and division [7]. Thus, deregulation of cellular energetics is a prominent cancer hallmark. Energy metabolism in cancer mainly includes glycolytic metabolism, as well as lipid and amino acid metabolism, of which only glycolytic metabolism has been reported to be regulated by circRNAs in thyroid cancers.

Ten circRNAs were shown to affect TC cell glycolytic metabolism directly or indirectly by regulating the expression of key aerobic glycolytic kinases. For example, hsa_circ_0004771 upregulates PKM2 expression via ceRNA mechanisms [82], and hsa_circ_0002111 phosphorylates PKM2 by upregulating FSTL1 [83] to promote glycolysis [72]. Likewise, hsa _circ_0122683 (circRAD18) [84] and hsa_circ_0001313 [85] directly upregulate PDK1 and PDK4, whereas hsa_circ_0122683 [37] and hsa_circ_0023990 [86] indirectly regulate PDKs to promote glycolysis [87, 88]. Additionally, the cell metabolic assays have demonstrated that hsa_circ_0000043 (circPUM1) [89] and hsa_circ_0058124 (circ0058124) [77] upregulate MAPK to promote glycolysis in thyroid cancers. However, the specific target molecule of glycolysis in MAPK remains unclear. No circRNAs were found to reprogram lipid or amino acid metabolism in thyroid cancers.

### Unlocking phenotypic plasticity

Unlocking phenotypic plasticity is restricted in normal cells but permitted in cancer cells, which enabling the emergence of diverse disrupted cell differentiation states that facilitate cancer initiation and progression. By exploring the interactions between circRNAs and differentiation-related genes, we may gain insights into the therapeutic challenges arising from the dedifferentiation of poorly differentiated, undifferentiated, and iodine-refractory thyroid cancers.

Dedifferentiation is a critical process in anaplastic ATC and represents the worst prognosis among thyroid cancers. ATC can undergo mutations independent of TCs at the gene mutation level, leading to a group of independent cancer cells. Notably, there are significant differences in the gene mutation profiles of ATC and papillary TC (PTC) [90], including mutations in *BRAF, RAS, TP53*, and *TERT*. These promote ATC’s ability to dedifferentiate and become more invasive [91]. CircRNAs may have a potentially promote TC differentiation or dedifferentiation through mutual regulation of differentiation-related tumor molecules. For example, in dedifferentiated TC cell lines, circ_0023990 regulates FOXM1 expression by competing with miR-485-5p to promote tumor growth and glycolysis. It has also been demonstrated that FOXM1,a transcription factor, plays a key role in cell dedifferentiation and promoting the acquisition of the EMT phenotype by lung adenocarcinoma stem cells [92].

Another example is circBACH2, which competes with miR-139-5p for LMO4 to promote the proliferation and invasion of PTC [93]. LMO4 is the only LIM transcription regulator family studied for its impact on the differentiation of breast [94] and oral cancers [95]. Therefore, circBACH2 may influence ATC cell differentiation.

Following dedifferentiation, radioiodine-refractory thyroid cancers lose expression of the Na/I symporter found in normal thyroid cells, rendering them unable to uptake iodine [96]. The differentiation level of PTC is associated with the aryl hydrocarbon receptor and its antagonist promotes PTC differentiation, although the exact mechanism is not fully understood. Reportedly, has_circ_0006741 promotes the expression of IGF2BP2 by regulating the sponge miR-4640-5p, leading to the overexpression of aryl hydrocarbon receptor protein in the nucleus, thereby promoting PTC dedifferentiation [97]. IGF2BP2 acts as an m6A reader, affects mRNA stability through post-transcriptional modifications, inhibits the proliferation, migration, and invasion of TC, and induces apoptosis and cell cycle arrest [98]. Further research is needed to investigate whether circRNA-mediated IGF2BP2 promotion contributes to PTC dedifferentiation and tumor proliferation.

### Non-mutational epigenetic reprogramming

Epigenetic reprogramming is a critical regulator of cancer onset and progression. This process includes DNA methylation, histone modification, nucleosome remodeling, and RNA modification [99–101]. RNA m6A modifications have been found to promote cancer cell growth, metastasis, metabolism, and drug resistance by enhancing circRNA expression and miRNA binding [87, 88, 102, 103]. Only one study of circRNA modifications in thyroid cancers has been published to date. In this study, knockout of ALKBH5, a demethylase involved in m6A modification, is significant. Its knockout enhances the expression of circNRIP1 such that miR-541-5p and miR-3064-5p are sponged, thereby jointly upregulating pyruvate kinase M2 expression and promoting glycolysis in TCs [82]. However, no correlation has yet been found between circRNAs and m6A modifications in thyroid cancers. Additionally, certain circRNAs can indirectly regulate DNA methylation or nuclear ribonucleoprotein, thereby promoting TC through non-mutational epigenetic reprogramming. For instance, hsa_circ_0061406 and hsa_circ_0007144 can respectively regulate the expression of HNRNPA1 and DNMT3A gene [104, 105]. Further research is needed to explore the potential roles of circRNA modifications in thyroid cancers and understand their contributions to epigenetic reprogramming.

## Prospects

**Fig. 2:**
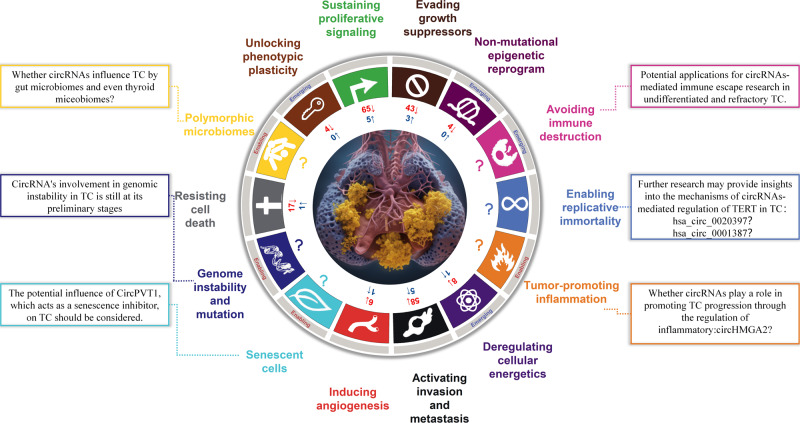
The review and prospects of circRNAs associated with thyroid cancer hallmarks. The figure provides an overview of circRNAs influencing thyroid cancer across different hallmarks. Numerical values indicate the quantity of circRNAs investigated for each hallmark. Red denotes promotion of thyroid cancer progression, blue indicates inhibition, and a question mark signifies no circRNA studies in this hallmark of thyroid cancer. Descriptions within the boxes outline prospects for further exploration.

Currently, research on the mechanisms of circRNA regulation in thyroid cancers has mainly focused on molecular interactions, with limited exploration of the mechanisms underlying circRNA biogenesis or degradation. Investigating whether circRNAs facilitate their production by modulating parental genes or regulating RnaseL to inhibit degradation remains worthwhile. In addition, considering the newest hallmarks, there is a significant gap in circRNA-related research specific tothyroid cancers, thus offering promising avenues for future exploration. To date, six cancer hallmarks have not been explored for circRNAs in thyroid cancers, see Table 1. Next, we briefly reviewed some genes, pathways, and circRNAs related to these six hallmarks in other cancers or diseases. This review may thus underscore the circRNAs worthy of study in thyroid cancers (Fig. 2).

### Avoiding immune destruction

Immune evasion is the phenomenon by which cancer cells evade immune surveillance, thereby promoting cancer progression. In thyroid cancers, immune escape mechanisms mainly involve the downregulation of autoantigen MHC molecules [106, 107] or the upregulation of PD-L1 [108, 109]. Additionally, thyroid cancers can downregulate the anti-tumor abilities [110] of immune cells and release cytokines [111] to reduce immune-mediated cell death. Some studies have shown that circRNAs can regulate tumor progression by avoiding immune evasion. Reportedly, circIGF2BP3 inhibits CD8 + T cell responses in non-small cell lung cancer [112], and circ_0020710 has been correlated with cytotoxic lymphocyte exhaustion and anti-PD-1 therapy resistance in melanoma [113]. However, current research on circRNA-mediated immune evasion in thyroid cancers is limited. Hence, there are potential applications for circRNA-mediated immune escape in undifferentiated and refractory thyroid cancers.

### Enabling replicative immortality

The maintenance of telomeric DNA is crucial for enabling the unlimited replication potential of cancer cells. The telomerase reverse transcriptase(TERT) gene, which encodes the telomerase catalytic subunit, can act as a cofactor to regulate various signaling pathways and is key in TC treatment [114]. Whole-transcriptome analysis has revealed that *TERT* and the telomerase RNA component are independent prognostic markers [115]. *TERT* mutations are strongly associated with non-radioiodine affinity in distant metastatically differentiated TC [116]. Some circRNAs, such as hsa_circ_0020397 [117] and hsa_circ_0001387(CircWHSC1) [118], promote the progression of rectal and ovarian cancers by upregulating *TERT*. However, the circRNAs that regulate thyroid cancers by targeting *TERT* remain unclear [119]. Further research could offer valuable insights into the mechanisms underlying the circRNA-mediated regulation of TERT in thyroid cancers. These findings may inform future therapeutic strategies that target telomerase activity during TC treatment.

### Tumor-promoting inflammation

Chronic inflammation is closely associated with a high incidence of cancer due to remodeling of the tumor microenvironment [120]. However, research on circRNAs that promote thyroid cancers via inflammation is lacking. Since circRNAs can influence the tumor microenvironment by regulating tumor angiogenesis and immune evasion [121, 122], functional circRNAs related to tumor-promoting inflammation should be identified. HMGB1, a late inflammatory factor, promotes TC progression [123]. One of these circRNAs, circHMGB2, has been found to promote cancer progression through inflammatory cells such as T cells, dendritic cells, and NK cells [124] in lung adenocarcinomas and squamous cell carcinomas. Moreover, circHMGA2, a circRNA derived from HMGA2, significantly promotes TC proliferation [125]. Consequently, it is important to investigate whether circHMGB2 and circHMGA2 also play similar roles in promoting TC progression by regulating the activity of inflammatory cells.

### Genome instability and mutation

Genomic instability and mutations in cancer have been extensively studied. However, the underlying mechanisms are diverse and complex. In addition to the G2/M DNA damage checkpoint, high frequencies of DNA damage and loss of repair proteins in the S phase contribute to genomic instability [96]. Certain circRNAs have been found to directly influence DNA damage repair and disease development [126, 127]. For instance, circRNA: DNA hybrids (circR loops) can trigger RNA polymerase stalling, leading to direct DNA damage that drives MLL gene recombination and promotes leukemia progression [128]. Moreover, circCIMT binds to APEX1 to mediate the DNA base excision repair pathway, thereby reducing DNA damage caused by cadmium [129]. Nevertheless, the involvement of circRNAs in genomic instability in thyroid cancers remains unclear, necessitating further exploration and investigation.

### Polymorphic microbiomes

Polymorphic microbiomes are increasingly being recognized as an enabling characteristic in cancer, potentially facilitating its initiation and progression. CircRNAs are known to influence diseases by affecting the gut microbiome. For example, circNF1-419 improves gut microbiome structure,benefitting an Alzheimer’s disease mouse model [130]. Studies have shown the presence of a microbiome in thyroid tissues, which were previously thought to be sterile [131–133]. The diversity and composition of the microbiome within and around TC tissues are significantly different [132] and can influence metabolism [133], progression, and invasion of thyroid cancers [131]. Thus, further investigations are warranted to explore whether circRNAs play a role in the interaction between the gut microbiome and TC. In addition, the Cancer-mbQTL database links genotypes from the Cancer Genome Atlas with Kraken-derived microbial data to analyze TC genetics and the microbiome [134], thus making it worthwhile to consider combining TC microbiome data with circRNA information.

### Senescent cells

Cellular senescence eliminates unwanted cells through tissue remodeling and has been developed as a pro-aging therapy to treat cancer and repair tissues [135, 136]. Although NF-κB, p38, mTOR, and C/EBPβ pathways promote cell senescence, only a limited number of circRNAs are involved in these pathways. Interestingly, CircPVT1 serves as a senescence inhibitor [137] in fibroblasts but promotes proliferation, invasion, resistance to cell death [61], and angiogenesis [53] in thyroid cancers. Whether circPVT1 plays an important role as a hallmark of senescent thyroid cancers remains unknown.

## Conclusion

TC poses significant challenges as the predominant cancer affecting the head and neck due to its diverse subtypes and complex mechanisms. CircRNAs have emerged as a promising area of cancer research and have excellent potential for both the diagnosis and treatment of thyroid cancers. In TC, eight hallmarks of cancer have been examined concerning circRNAs, while the other six hallmarks have not yet been studied. Most of these circRNAs are related to tumor proliferation, making them potential biomarkers for TC. The future holds promise in elucidating the potential role of circRNAs in the regulation of inflammation in TC through tumor-promoting inflammation. Additionally, exploring drugs such as PD-L1 to prevent immune destruction, holds potential for treating undifferentiated TC by circRNAs [112, 117]. Not only in terms of hallmarks, but limitations also exist in the pathological types (less PDTC or ATC) and molecular interaction mechanisms (mostly ceRNA), providing new starting points for further in-depth research. This review is expected to contribute to the understanding and application of circRNAs in precise diagnostic approaches and the development of innovative therapeutic strategies for thyroid cancers.

### Supplementary information

Categorization based on hallmarks: comprehensive researchs of circRNA mechanisms in thyroid cancer
